# Achieving high spatial and temporal resolution with perfusion MRI in the head and neck region using golden-angle radial sampling

**DOI:** 10.1007/s00330-020-07263-0

**Published:** 2020-09-24

**Authors:** Andrea Tomppert, Wolfgang Wuest, Marco Wiesmueller, Rafael Heiss, Markus Kopp, Armin M. Nagel, Hayato Tomita, Christian Meixner, Michael Uder, Matthias Stefan May

**Affiliations:** 1 Department of Radiology, University Hospital Erlangen, Maximiliansplatz 3, 91054 Erlangen, Germany; 2 Imaging Science Institute, University Hospital Erlangen, Erlangen, Germany; 3 Institute of Medical Physics, Friedrich-Alexander-Universität Erlangen-Nürnberg (FAU), Erlangen, Germany; 4 Department of Radiology, St. Marianna University School of Medicine, Miyamae-ku, Kawasaki, Japan

**Keywords:** Adult, Magnetic resonance imaging, Neck, Perfusion

## Abstract

**Objectives:**

Conventional perfusion-weighted MRI sequences often provide poor spatial or temporal resolution. We aimed to overcome this problem in head and neck protocols using a golden-angle radial sparse parallel (GRASP) sequence.

**Methods:**

We prospectively included 58 patients for examination on a 3.0-T MRI using a study protocol. GRASP (A) was applied to a volumetric interpolated breath-hold examination (VIBE) with 135 reconstructed pictures and high temporal (2.5 s) and spatial resolution (0.94 × 0.94 × 3.00 mm). Additional sequences of matching temporal resolution (B: 2.5 s, 1.88 × 1.88 × 3.00 mm), with a compromise between temporal and spatial resolution (C: 7.0 s, 1.30 × 1.30 × 3.00 mm) and with matching spatial resolution (D: 145 s, 0.94 × 0.94 × 3.00 mm), were subsequently without GRASP. Instant inline-image reconstructions (E) provided one additional series of averaged contrast information throughout the entire acquisition duration of A. Overall diagnostic image quality, edge sharpness and contrast of soft tissues, vessels and lesions were subjectively rated using 5-point Likert scales. Objective image quality was measured as contrast-to-noise ratio in D and E.

**Results:**

Overall, the anatomic and pathologic image quality was substantially better with the GRASP sequence for the temporally (A/B/C, all *p* < 0.001) and spatially resolved comparisons (D/E, all *p* < 0.002 except lesion edge sharpness with *p* = 0.291). Image artefacts were also less likely to occur with GRASP. Differences in motion, aliasing and truncation were mainly significant, but pulsation and fat suppression were comparable. In addition, the contrast-to-noise ratio of E was significantly better than that of D (*p*_D-E_ < 0.001).

**Conclusions:**

High temporal and spatial resolution can be obtained synchronously using a GRASP-VIBE technique for perfusion evaluation in head and neck MRI.

**Key Points:**

*• Golden-angle radial sparse parallel (GRASP) sampling allows for temporally resolved dynamic acquisitions with a very high image quality.*

*• Very low-contrast structures in the head and neck region can benefit from using the GRASP sequence.*

*• Inline-image reconstruction of dynamic and static series from one single acquisition can replace the conventional combination of two acquisitions, thereby saving examination time.*

**Electronic supplementary material:**

The online version of this article (10.1007/s00330-020-07263-0) contains supplementary material, which is available to authorized users.

## Introduction

Perfusion MRI has become increasingly important in distinguishing malignant from benign lesions, particularly in the head and neck region [1, 2]. The problem of dynamic contrast-enhanced (DCE) MRI is that there is a trade-off between high temporal or high spatial resolution. Fast signal intensity changes must be observed in a short time during the passage of the contrast agent, but anatomical information has to be sufficiently detailed to evaluate the location in relation to the surrounding tissues. Therefore, conventional perfusion protocols in head and neck MRI include a temporally resolved DCE sequence with rather poor spatial resolution during contrast injection and a subsequent spatially resolved acquisition [3]. The disadvantage of the frequently used Cartesian T1-weighted sequences with fat suppression is that they provide either high temporal or high spatial resolution. Hence, a compromise of intermediate temporal and spatial resolution is often selected for DCE sequences in a routine clinical setting [4, 5]. Compressed sensing (CS) techniques recently opened up several opportunities for accelerating MRI [6, 7]. One approach of CS is the radial sampling of k-space. In comparison to standard sampling of k-space, where the data is sampled on a Cartesian grid, radial sampling generates data points that do not fit into a rectangular matrix. This radially acquired data has to be morphed into a Cartesian matrix during post-processing.

Winkel et al showed that CS radial sampling perfusion MRI is able to improve diagnostic accuracy in prostate cancer when combined with diffusion-weighted imaging sequences [8]. In addition, Feng et al in their conceptional study using iterative golden-angle radial sparse parallel (GRASP) MRI proved a high level of clinical performance and flexibility because of ruggedness to motion by simultaneous acquisition of high spatial and temporal resolution [9].

Recently, a commercially available GRASP sequence with automated post-processing on the MRI console was released, meaning that implementation of this relatively new technological approach into a clinical workflow now seems achievable.

Our aim in this study was to evaluate whether the simultaneous acquisition of high spatial and temporal resolution is feasible by GRASP implementation into clinical head and neck protocols. Conventional techniques served as a reference for our non-inferiority hypothesis, which stated that image quality from a single GRASP acquisition can be comparable with the two previously established measurements.

## Materials and methods

### Intra-patient comparison

**Fig. 1:**
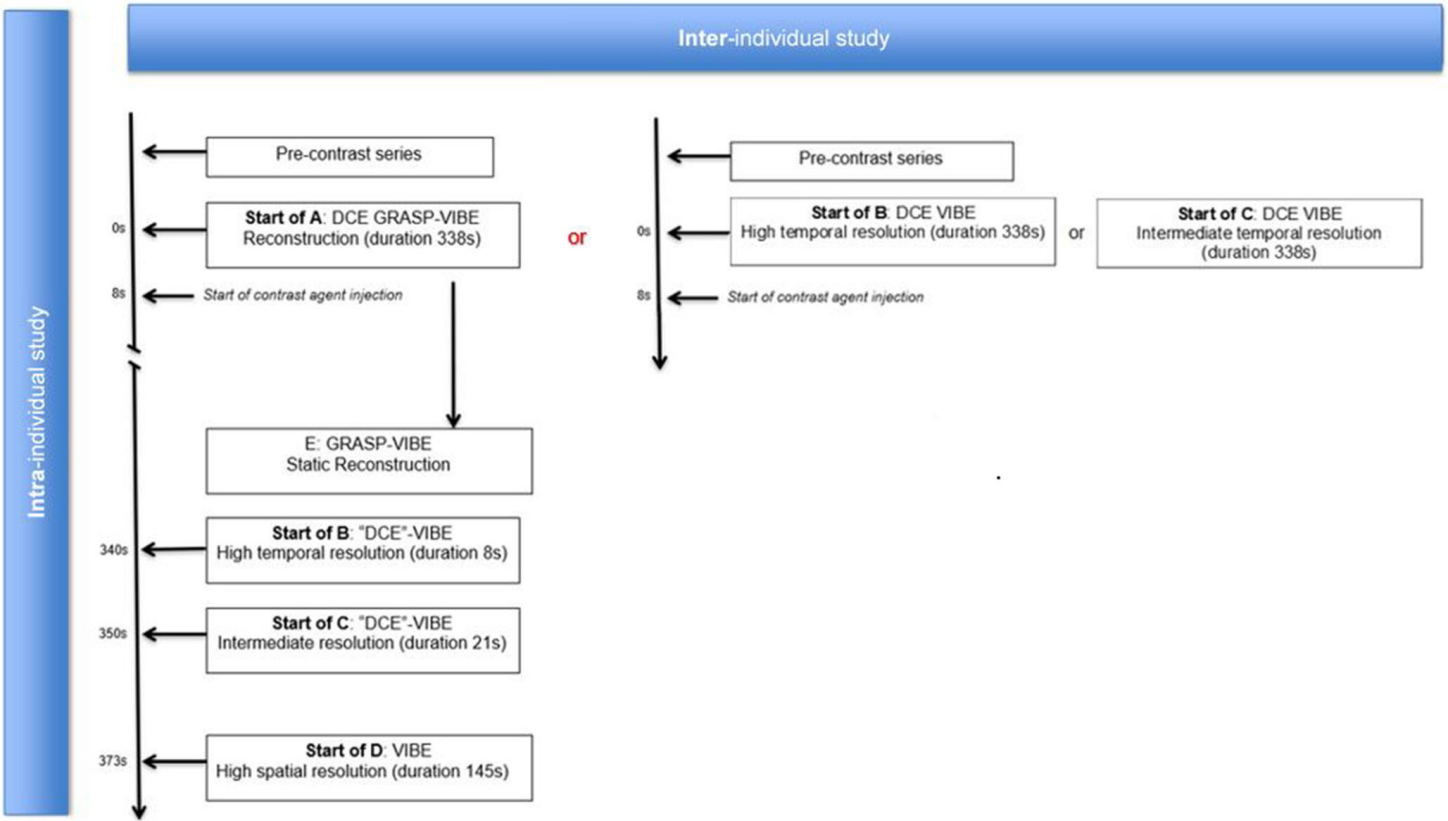
Flowchart of study design. Golden-angle radial sparse parallel (GRASP), volumetric interpolated breath-hold examination (VIBE), dynamic contrast-enhanced (DCE)

During the study conception, a sample size calculation was performed after evaluation of the first ten patients following a non-inferiority hypothesis for overall image quality and a desired statistical power of 95%. Exclusion criteria were general contraindications for an MRI examination (i.e. non-MRI conditional pacemaker, non-medical metal fragments or implants, claustrophobia), contraindications for intravenous contrast agent injection (prior allergic reactions, high-grade renal insufficiency) or a refusal to participate. From May 2018 to January 2019, a total of 58 patients with indications for head and neck MRI were included and consecutively scheduled on a 3.0-T MRI system (Magnetom Vida, Siemens Healthcare GmbH) for the intra-patient image evaluation (Fig. 1). The study complies with the Declaration of Helsinki, institutional review board approval was obtained, and all patients gave their informed consent in writing.

### Inter-patient comparison

An intra-individual comparison of DCE sequences with repetitive contrast agent injections is not justifiable due to ethical reasons. Moreover, it is unlikely that DCE with repetitive contrast injections would provide comparable results, because of the contrast-saturated interstitial space left over from the previous exam. Therefore, in order to obtain also a dynamic comparison between A, B and C, an additional inter-patient evaluation was performed. From September to December 2019, consecutive patients were randomised to examinations using sequence B or C as the DCE sequence (Fig. 1). The exclusion criteria and ethical considerations remained the same.

### MRI technique

All patients underwent contrast-enhanced imaging on a 3.0-T MR scanner using a 64-channel head and neck coil. Pre-contrast series were obtained following the institutional reference protocol. Bodyweight-adapted (0.01 mmol/kg body weight) doses of gadobutrol contrast agent (Gadovist, Bayer) were administered at 2 ml/s using a dedicated power injector (Accutron MR3, Medtron AG), followed by a 30 ml saline flush (0.9%) administered with the same injection speed. The golden-angle radial sparse parallel (GRASP) technique (Siemens Healthcare GmbH) was applied to a transversal T1-weighted volume-interpolated gradient-echo perfusion sequence (VIBE), which was started 8 s prior to contrast injection (A). Golden-angle ordering means that each spoke is incremented by 111.25°, which is 180° divided by the golden ratio (approximately 1.62) [10]. A total of 135 reconstructed picture series were obtained for each patient with the minimum temporal offset available (2.5 s) over 338 s of total acquisition time (TR 4.09 ms, TE 1.95 ms, flip angle 12°, bandwidth 500 Hz/pixel).

Voxel size was adapted to the institutional T1-weighted fat-saturated reference sequence after contrast injection (0.94 × 0.94 × 3.00 mm), which is comparable with datasets in the literature [8]. The inline-image reconstruction technique provides one series (E) of averaged contrast information over the entire acquisition duration (STATIC, Siemens Healthcare GmbH) and a stack of series for every point in time of the dynamics (135 series in one stack, A). Additional transversal sequences using the conventional technique were subsequently acquired with matching temporal resolution (B), with a trade-off in parameters between temporal and spatial resolution (C) and with matching spatial resolution (D). Detailed sequence information is given in Table 1. Sequences B and C were run over a period of three images (8 and 21 s) for intra-individual comparison, and over a period of 338 s for inter-individual comparison.

**Table 1:** Technical parameters of the study (A) and reference sequences (B–D). Reconstructed image series and total acquisition time of sequences B and C refer to the intra-patient comparison

	A	B	C	D	E
Sampling	GRASP	Cartesian	Cartesian	Radial	GRASP
Temporal resolution (s)	2.5	2.5	7.0	145.0	338.0
In-plane resolution (mm)	0.94 × 0.94	1.88 × 1.88	1.30 × 1.30	0.94 × 0.94	0.94 × 0.94
Slice (mm)	3.00	3.00	3.00	3.00	3.00
Reconstructed image series	135	3	3	1	1
Repetition time TR (ms)	4.09	4.18	4.18	4.09	4.09
Echo time TE (ms)	1.95	1.6	1.77	1.95	1.95
Flip angle (deg)	12	12	12	12	12
Total acquisition time (s)	338	8	21	145	338
Bandwidth (Hz/pixel)	501	501	501	501	501

### Image evaluation

Two experienced radiologists (5 years’ and 10 years’ experience, respectively) evaluated the subjective image quality as overall diagnostic image quality, soft tissue edge sharpness, soft tissue contrast, vessel edge sharpness, vessel contrast, lesion edge sharpness and lesion contrast. Artefacts were divided into sequence-dependent artefacts such as truncation, aliasing or insufficient fat suppression, as well as patient-dependent artefacts such as pulsation artefacts of blood vessels or motion artefacts, e.g. swallowing movements. Overall, anatomical and pathological image quality parameters were rated on a 5-point Likert scale, as shown in Table 2. Subjective image quality was assessed for the intra- and inter-individual comparisons, while objective image quality was assessed for the intra-individual comparisons.

**Table 2:** Detailed evaluation criteria for each image evaluation parameter

Parameter	Score	Criteria
Overall image quality	1	Unevaluable
	2	Limited diagnostic information
	3	Acceptable diagnostic information
	4	Adequate diagnostic information
	5	Definite diagnostic information
Soft tissue/vessel edge sharpness	1	No margins to surrounding tissues
	2	Doubtful margins
	3	Blurred margins
	4	Sharp margins
	5	Very sharp margins
Soft tissue/vessel contrast	1	No contrast
	2	Doubtful contrast
	3	Slight contrast
	4	Good contrast
	5	Significant contrast
Artefacts	1	Very strong artefacts
	2	Strong artefacts
	3	Medium artefacts
	4	Small artefacts
	5	Negligible artefacts

The objective image quality was assessed as signal-to-noise ratio (SNR) and contrast-to-noise ratio (CNR) between the left common carotid artery (CCA) and the left levator scapulae muscle (LSM) in the image series with high spatial resolution (D and E) following Eqs. 1 and 2:

1 $$ \mathrm{SNR}={\mathrm{Mean}}_{\mathrm{CCA}}/{\mathrm{Noise}}_{\mathrm{LSM}} $$

2 $$ \mathrm{CNR}=\left({\mathrm{Mean}}_{\mathrm{CCA}}-{\mathrm{Mean}}_{\mathrm{LSM}}\right)/{\mathrm{Noise}}_{\mathrm{LSM}} $$

**Fig. 2:**
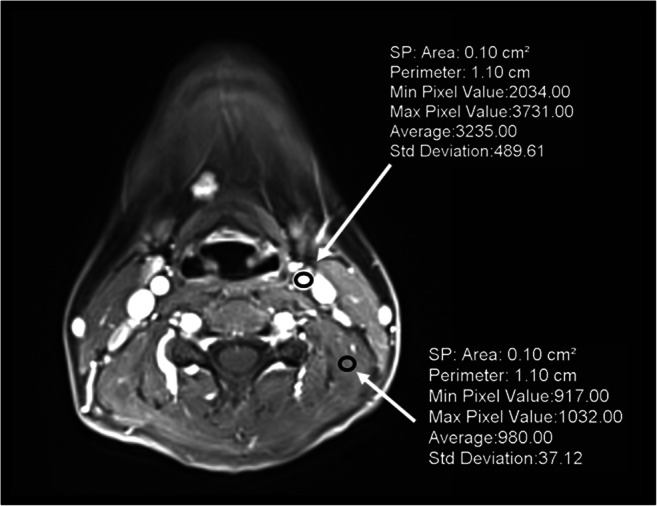
Series of averaged contrast information over the entire acquisition duration (E) with regions of interest in the left common carotid artery (CCA) and levator scapulae muscle (LSM) to calculate the image contrast

Signal intensities were measured by placing regions of interest as shown in Fig. 2.

### Statistics

All statistical tests were performed using SPSS, Version 21 (IBM). Values are presented as mean and standard deviation if normally distributed, and as median and range if not. A non-parametric Friedman test with post hoc tests (Dunn-Bonferroni test) was performed in order to compare the temporally resolved image series (A–C), and a non-parametric Wilcoxon rank-sum test was performed to compare the spatially resolved image series (D and E). Inter-patient evaluation was performed using a Kruskal-Wallis test. *p* values below 0.05 were considered to be statistically significant.

## Results

### Intra-patient comparison

The sample size calculation required a minimum of 47 patients, so our sample size of 58 patients was adequate. The mean age of the 58 patients (36 female and 22 male) was 53 (ranging from 19 to 86 years). The median of injected contrast agent was 8 ml (range 6–15 ml) per patient. The indication for the procedure was for differential diagnosis of suspicious lesions in 43 patients (74.1%) and follow-up after surgical resection in 15 patients (25.9%). Pathologic lesions were found in all of the study examinations, 40 patients (69%) had a single lesion and 18 patients (31%) had multiple lesions. However, malignant lesions were found in only nine patients (16%). In each case, only the main lesion was evaluated. The median lesion size was 2 cm (range 0.6–4.7 cm), and scar tissue was considered as a non-measurable lesion. A surgical resection of the lesions was performed in 25 patients following an interdisciplinary board decision. The histopathological results are shown in Table 3. In 86% of cases, the MRI findings were consistent with the histological findings. The sensitivity (100%) and specificity (89%) for the differentiation of malignant and benign lesions were either comparable with or slightly superior to the values in the literature [11–14]. Discrepancies between the MRI findings and the histological analysis were found in two cases, where two adenomas had been wrongly classified as malignant tumours.

**Table 3:** Histopathological results of lesions resected based on the basis of the MRI findings

Histopathology	*n* = 25
Adenoma of the parotid gland	11
Carcinoma	6
Warthin tumour	3
Cyst of the parotid gland	2
Reactive lymph node (benign)	2
Lymphoma	1

### Inter-patient comparison

An interim evaluation was performed after inclusion of 12 patients who have had a DCE examination using series B, and 14 patients who have had a DCE examination using series C. The overall image quality of the GRASP-VIBE sequence was significantly better compared with both, B (*p* < 0.001) and C (*p* < 0.001). These clear differences confirmed the intra-individual evaluation (see also Electronic Supplementary Material). H; hence, the additional inter-patient study was discontinued for ethical reasons.

### Image quality

The inter-observer agreement was good (overall *κ* = 0.76, range 0.68–1.00). The three temporally resolved datasets were significantly different for the overall image quality and for all of the anatomical and pathological evaluations (*p* < 0.001).The highest ranks and almost always excellent ratings were obtained for A (Table 4). All post hoc comparisons were significantly different, and highest rank differences were found for overall image quality (*p*_A-B_ and *p*_A-C_ < 0.001), soft tissue edge sharpness (*p*_A-B_ and *p*_A-C_ < 0.001) and vessel edge sharpness (*p*_A-B_ and *p*_A-C_ < 0.001). Differences in contrast (soft tissue, vessel and lesion) were also significant, but with lower rank differences (*p*_A-B_ and *p*_A-C_ < 0.001). The clinically established trade-off sequence (C) was ranked between A and B in all categories.

**Table 4:** Median values of subjective image quality evaluation and *p* values for the relevant comparisons. Range is shown in brackets; significant values are presented in italics

	A	B	*p* (A–B)	C	*p* (A–C)	D	E	*p* (D–E)
Overall image quality	5 (3–5)	2 (2–3)	*< 0.001*	3 (3–4)	*< 0.001*	4 (3–5)	5 (3–5)	*< 0.001*
Soft tissue edge sharpness	5 (3–5)	2 (2–3)	*< 0.001*	3 (3–4)	*< 0.001*	4 (3–5)	4 (3–5)	*< 0.002*
Soft tissue contrast	4 (3–5)	4 (2–5)	*< 0.001*	4 (2–5)	*< 0.001*	4 (3–5)	5 (4–5)	*< 0.001*
Vessel edge sharpness	5 (4–5)	2 (2–3)	*< 0.001*	3 (3–4)	*< 0.001*	4 (3–5)	4 (3–5)	*< 0.002*
Vessel contrast	5 (4–5)	3 (2–4)	*< 0.001*	4 (3–5)	*< 0.001*	4 (3–5)	5 (5)	*< 0.001*
Lesion edge sharpness	5 (2–5)	3 (1–3)	*< 0.001*	3 (1–4)	*< 0.001*	5 (1–5)	5 (3–5)	0.291
Lesion contrast	5 (1–5)	4 (1–5)	*< 0.001*	4 (1–5)	*< 0.001*	4 (1–5)	5 (1–5)	*< 0.001*
Motion artefacts	4 (3–5)	4 (3–5)	*< 0.001*	4 (2–5)	*< 0.001*	4 (3–5)	4 (4–5)	*< 0.001*
Aliasing artefacts	4 (3–4)	4 (2–4)	0.414	4 (4)	*< 0.001*	4 (2–5)	4 (4–5)	*< 0.001*
Truncation artefacts	4 (4–5)	4 (2–5)	*< 0.001*	4 (3–5)	*< 0.001*	3 (3–4)	4 (4–5)	*< 0.001*
Pulsation art facts	5 (4–5)	5 (3–5)	0.368	5 (3–5)	0.368	5 (5)	5 (5)	1.000
Fat suppression artefacts	4 (3–5)	4 (3–5)	0.564	4 (3–5)	0.317	4 (2–5)	4 (2–5)	*< 0.001*

*A* dynamic GRASP reconstructions, *B* temporally resolved reference, *C* trade-off with intermediate temporal and spatial resolution, *D* spatially resolved reference, *E* static GRASP reconstruction

Motion artefacts and truncation artefacts were significantly less prominent in A (*p*_A-B_ and *p*_A-C_ < 0.001). Aliasing artefacts in the GRASP acquisition (A) were comparably frequent as in B (*p*_A-B_ = 0.414) and not as prominent but were significantly more frequent compared with C (*p*_A-C_ < 0.001). Artefacts of fat suppression (*p*_A-B_ = 0.564 and *p*_A-C_ = 0.317) had no significant difference. Neither A, B nor C had any pulsation artefacts. All cases of limited fat suppression were detected in the shoulder area. Examples for dynamic contrast perfusion measured with A–C are shown in the supplemental digital material.

**Fig. 3:**
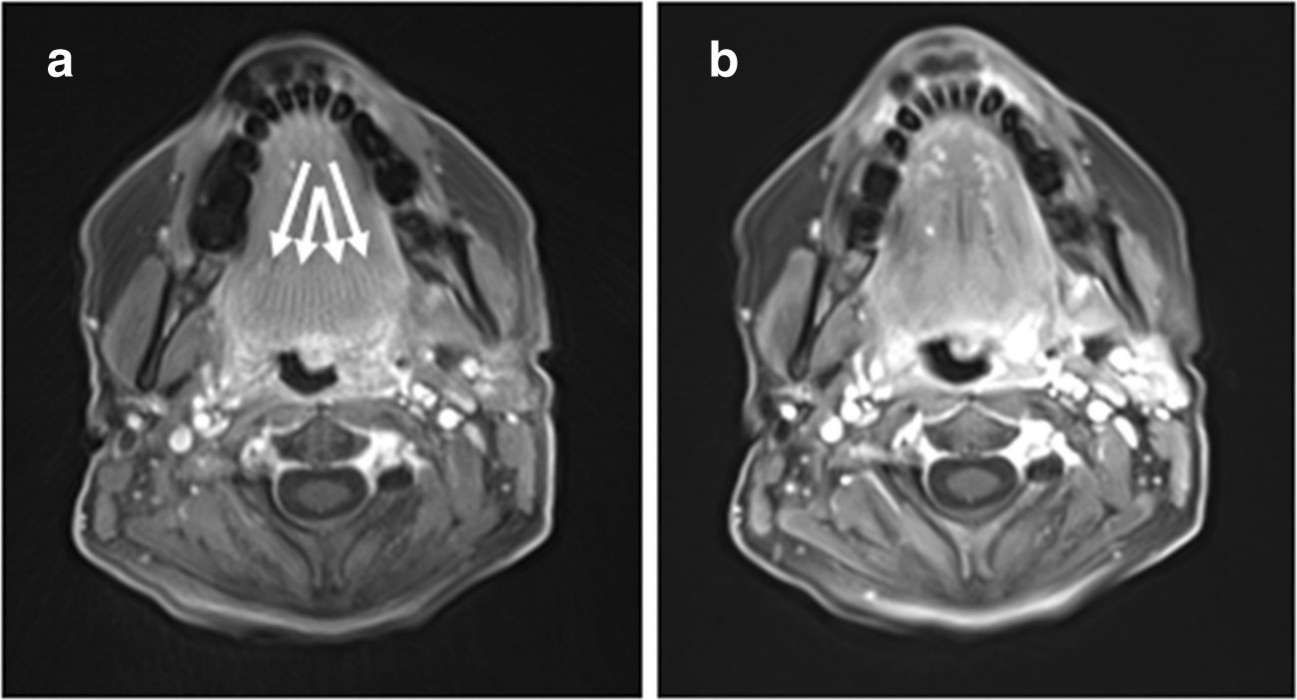
Aliasing artefacts are more intense in (**a**) compared with the static GRASP reconstruction (**b**)

**Fig. 4:**
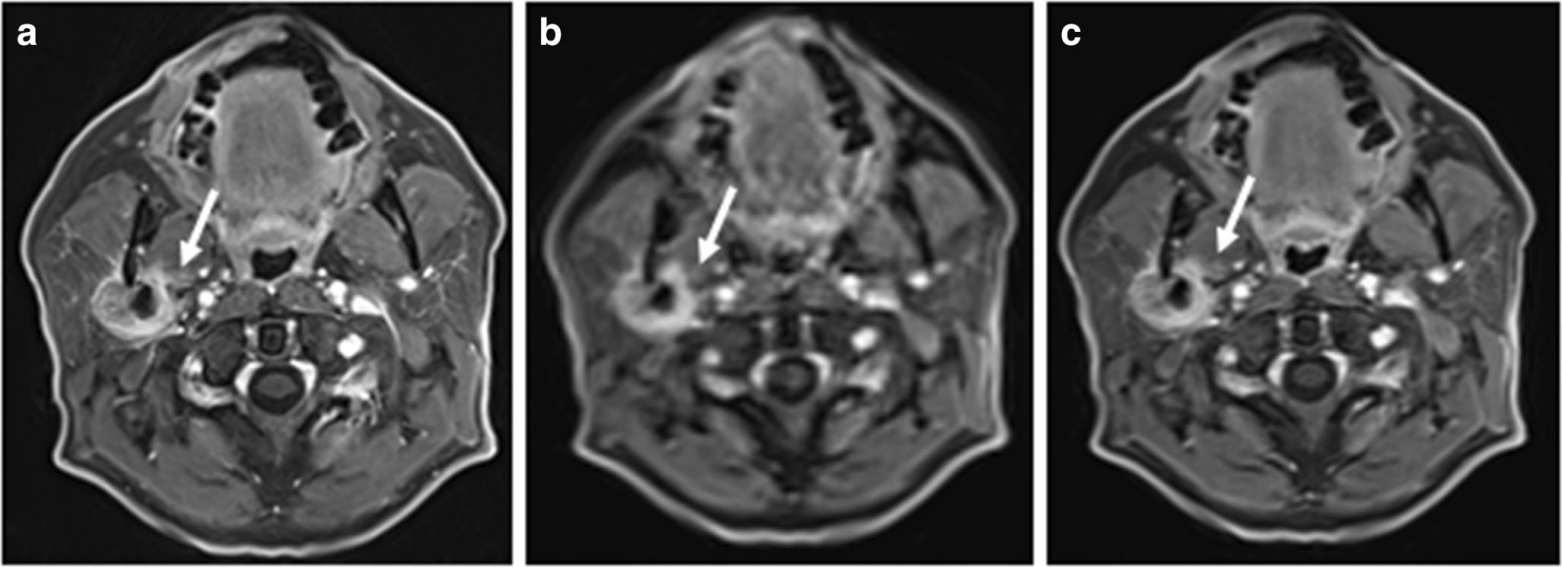
A 56-year-old man with histologically proven parotid adenocarcinoma. The GRASP acquisition (**a**: 2.5 s) has a better sharpness of anatomical structures and enhancement of, e.g. mucosal folds when compared with **b** (2.5 s) and **c** (7.0 s). Infiltration of the right pterygoid muscle and the carotid space is best reproduced in **a** (arrows)

**Fig. 5:**
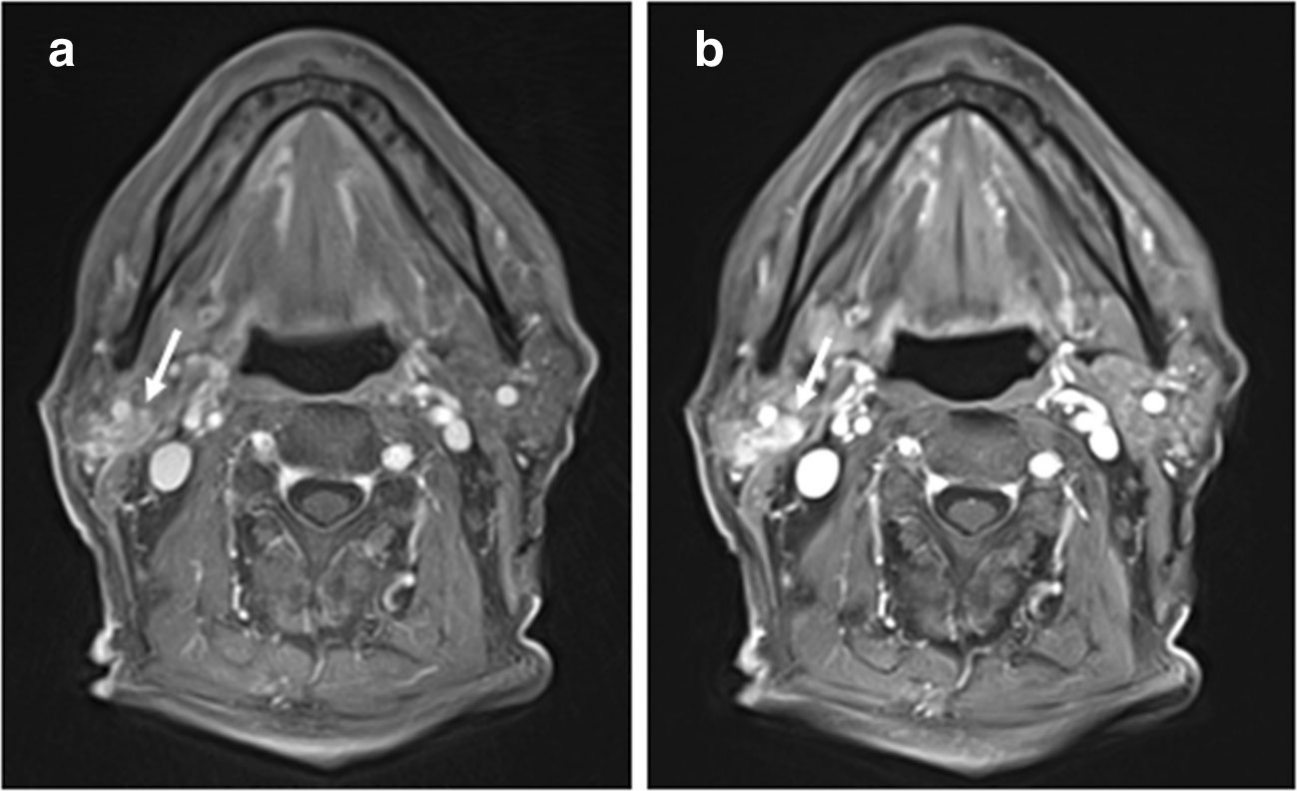
An 86-year-old man with a histologically proven Warthin tumour in the right parotid gland. The reconstructed GRASP series (**a**) shows a better soft tissue, vessel and lesion contrast (arrows) than the VIBE (**b**) with matching spatial resolution

**Fig. 6:**
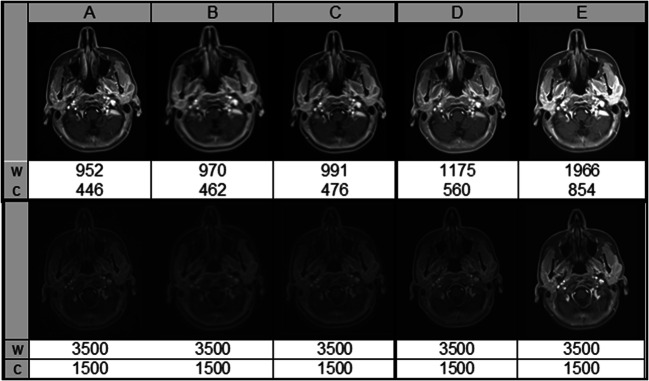
A 25-year-old woman with a histologically proven inflammatory reactive lymph node in the left parotid gland. Images are shown in the default window (top row) as provided by the scanner (w: width, c: centre) and in a window adapted to the high signal intensities of the static GRASP reconstruction (E). The SNR/CNR values for A–E (top row) were as follows: A 144/109, B 95/69, C 139/103, D 120/89, E 194/144. SNR and CNR of A–E were not affected by the window adaption (lower row)

Differences between the static reconstruction of the GRASP sequence (E) and the spatially resolved reference (D) were small but statistically significant for most of the evaluated criteria, with higher ranks for E. The overall image quality, contrast (soft tissue, vessel, lesion) and most artefacts (motion, aliasing, truncation, fat suppression) were rated as significantly better (*p*_D-E_ < 0.001) in the study sequence (Fig. 3). The differences between soft tissue edge sharpness and vessel edge sharpness were slightly less but were still significant (*p*_D-E_ < 0.002). Lesion edge sharpness was also comparable (*p*_D-E_ = 0.291) and neither D nor E had any pulsation artefacts (*p*_D-E_ = 1.000). All median values of the subjective image quality evaluation and all *p* values of the intra-patient comparison are provided in Table 4, with image examples shown in Figs. 4 and 5. Example pictures of one patient with optimal windowing for each sequence (A–E) as well as with identical windowing for each sequence (A–E) are shown in Fig. 6.

The objective image quality of E was significantly better than of D (*p*_D-E_ < 0.001). The median SNR was 200 (range 58–823) in D and 415 (range 124–2904) in E, while the median CNR was 135 (range 36–586) in D and 273 (range 81–1986) in E.

## Discussion

Dynamic and static reconstructions from GRASP-VIBE acquisitions provided equal or even superior image quality in comparison with the conventional technique. The intra-individual results of this study are confirmed by the inter-individual assessment, which was terminated due to highly significant and ethically unjustifiable differences in the interim evaluation. Dynamic GRASP reconstructions can substantially improve image quality in low-contrast structures like the soft tissues of the head and neck region. This could potentially also favour the diagnostic accuracy of low-contrast lesions, which hence should be systematically evaluated in further controlled studies. We also found improved image quality in the static reconstructions of GRASP acquisitions (E) when compared with the conventional technique with a similar voxel size (D). This could be caused by a combination of a higher signal due to longer acquisition, lower noise due to CS and improved contrast due to first-pass acquisition of the contrast agent. This was particularly beneficial in cases of soft tissue, vessel and lesion contrast. To our knowledge, no previous study has compared this static reconstruction from the GRASP acquisition. Our results, therefore, contribute to enrich the knowledge surrounding the diverse benefits from combining CS and golden-angle radial sampling beyond the current state of the literature [2]. The improved image quality of DCE reconstructions from GRASP acquisitions in the head and neck region is in good agreement with studies from other regions such as the head, lungs, breast or liver [15–18]. For example, Sen et al demonstrated that the localisation of macroadenomas of the pituitary gland can be improved, which is a very important preoperative step [15]. Rosenkrantz et al also demonstrated improvements in image quality and lesion depiction of DCE-GRASP [19]. According to Heacock et al, GRASP DCE-MRI has a near comparable performance with conventional VIBE imaging for breast lesion evaluation, which is also in line with our findings [17].

Additionally, functional assessment such as the evaluation of tumour angiogenesis in lung cancer was shown by Chen et al [20]. Extended benefits like the functional assessment for rectal cancer, as shown by Attenberger et al, could be feasible for the head and neck region as well and should be evaluated in further studies with histopathological correlations [21]. The GRASP-VIBE sequence was also consistent with regard to the number of artefacts. None of the observed artefacts affected the assessment in the relevant regions. Minor aliasing artefacts were found in all sequences, but in different spreads. The GRASP technique had typical wraparound aliasing artefacts of radial and spiral sampling with curvilinear stripes, whereas the Cartesian sequences had the typical aliasing of rectilinear scanning. The GRASP technique exhibited very few motion artefacts, which correlates with the study results of Riffel et al [22].

GRASP-VIBE is assessed as a single acquisition, while conventional techniques require two separate acquisitions to obtain high temporal and spatial resolution. Hence, a single GRASP sequence could also replace the traditional combination of series, as demonstrated in the “one-stop-shop” approach of Riffel et al for the kidneys. The relatively long reconstruction time of around 5 min may reduce this benefit, but the mere time of measurement with the patient in the scanner could be reduced by around 2 to 3 min. The maximum temporal resolution of the GRASP technique of 2.5 s is also comparable or even higher than that of time-resolved MR angiographies (e.g. TWIST) [23].

Some limitations of this study merit consideration. First, patients were randomly selected meaning that mixed pathologies are included in the evaluation of lesion delineation. The good results, especially for the low-contrast structures and lesion contrast compared with the conventional technique, strongly support further studies with dedicated evaluations of pathologies and performance calculation.

Second, for ethical reasons, we were not able to provide an intra-individual comparison of DCE. Therefore, we had to limit our systematic evaluation to image quality features, so the influence of dynamic parameters such as curve analysis remains unclear. Following the positive results of Winkel et al [8] for prostate cancer, further studies to evaluate the impact of an improved temporal resolution of only 2.5 s are also encouraged. Third, the availability of the GRASP technique is still limited today. However, we are convinced that the advantages of such four-dimensional sequences will be used more widely in routine clinical procedures. Fourth, only mainly subjective image features were evaluated for this study because objective parameters are limited by comparability between different acquisition techniques. We sought to overcome this limitation with the CNR and SNR calculations between the carotid arteries and the muscles in D and E. Finally, a large amount of data was created that required high computational power for post-processing, which may not yet be available into all radiology departments.

## Conclusion

The simultaneous acquisition of high spatial and temporal resolution is feasible for contrast-enhanced head and neck perfusion MRI by the application of the GRASP technique. These four-dimensional sequences are able to increase diagnostic image quality and perfusion information, which seems particularly advantageous in the soft tissues of the head and neck region. Results can be obtained by inline reconstruction in the latest scanner generations, making it applicable in routine clinical workflow.

## Electronic supplementary material

ESM 1 (WMV 192 kb)

ESM 2 (WMV 197 kb)

ESM 3 (WMV 180 kb)

## Abbreviations

CCA: Common carotid artery
CNR: Contrast-to-noise ratio
CS: Compressed sensing
DCE: Dynamic contrast-enhanced
GRASP: Golden-angle radial sparse parallel
LSM: Levator scapulae muscle
SNR: Signal-to-noise ratio
STATIC: Static reconstruction of GRASP technique
TWIST: Time-resolved angiography with interleaved stochastic trajectories
VIBE: Volumetric interpolated breath-hold examination

## References

[1] Mikaszewski B, Markiet K, Smugała A, Stodulski D, Szurowska E, Stankiewicz C (2017). Diffusion- and perfusion-weighted magnetic resonance imaging–an alternative to fine needle biopsy or only an adjunct test in preoperative differential diagnostics of malignant and benign parotid tumors?. J Oral Maxillofac Surg.

[2] Choi SH, Lee JH, Choi YJ (2017). Detection of local tumor recurrence after definitive treatment of head and neck squamous cell carcinoma: histogram analysis of dynamic contrast-enhanced T1-weighted perfusion MRI. AJR Am J Roentgenol.

[3] Yuan J, Chow SK, Yeung DK, King AD (2012). A five-colour colour-coded mapping method for DCE-MRI analysis of head and neck tumours. Clin Radiol.

[4] Yuan Y, Shi H, Tao X (2016). Head and neck paragangliomas: diffusion-weighted, and dynamic contrast-enhanced magnetic resonance imaging characteristics. BMC Med Imaging.

[5] Park M, Kim J, Choi YS (2016). Application of dynamic contrast-enhanced MRI parameters for differentiating squamous cell carcinoma and malignant lymphoma of the oropharynx. AJR Am J Roentgenol.

[6] Lustig M, Donoho D, Pauly JM (2007). Sparse MRI: the application of compressed sensing for rapid MR imaging. Magn Reson Med.

[7] Feng L, Benkert T, Block KT, Sodickson DK, Otazo R, Chandarana H (2017). Compressed sensing for body MRI. J Magn Reson Imaging.

[8] Winkel DJ, Heye TJ, Benz MR (2019). Compressed sensing radial sampling MRI of prostate perfusion: utility for detection of prostate cancer. Radiology.

[9] Feng L, Grimm R, Block KT (2014). Golden-angle radial sparse parallel MRI: combination of compressed sensing, parallel imaging, and golden-angle radial sampling for fast and flexible dynamic volumetric MRI. Magn Reson Med.

[10] Winkelmann S, Schaeffter T, Koehler T, Eggers H, Doessel O (2007). An optimal radial profile order based on the golden ratio for time-resolved MRI. IEEE Trans Med Imaging.

[11] Christe A, Waldherr C, Hallett R, Zbaeren P, Thoeny H (2011). MR imaging of parotid tumors: typical lesion characteristics in MR imaging improve discrimination between benign and malignant disease. AJNR Am J Neuroradiol.

[12] van der Hoorn A, van Laar PJ, Holtman GA, Westerlaan HE (2017). Diagnostic accuracy of magnetic resonance imaging techniques for treatment response evaluation in patients with head and neck tumours: a systematic review and meta-analysis. PLoS One.

[13] Yerli H, Aydin E, Haberal N, Harman A, Kaskati T, Alibek S (2010). Diagnosing common parotid tumours with magnetic resonance imaging including diffusion-weighted imaging vs fine-needle aspiration cytology: a comparative study. Dentomaxillofac Radiol.

[14] Vandecaveye V, De Keyzer F, Nuyts S (2007). Detection of head and neck squamous cell carcinoma with diffusion-weighted MRI after (chemo)radiotherapy: correlation between radiologic and histopathologic findings. Int J Radiat Oncol Biol Phys.

[15] Sen R, Sen C, Pack J (2017). Role of high-resolution dynamic contrast-enhanced MRI with golden-angle radial sparse parallel reconstruction to identify the normal pituitary gland in patients with macroadenomas. AJNR Am J Neuroradiol.

[16] Chen L, Liu D, Zhang J (2018). Free-breathing dynamic contrast-enhanced MRI for assessment of pulmonary lesions using golden-angle radial sparse parallel imaging. J Magn Reson Imaging.

[17] Heacock L, Gao Y, Heller SL (2017). Comparison of conventional DCE-MRI and a novel golden-angle radial multi-coil compressed sensing method for the evaluation of breast lesion conspicuity. J Magn Reson Imaging.

[18] Chandarana H, Feng L, Ream J (2015). Respiratory motion-resolved compressed sensing reconstruction of free-breathing radial acquisition for dynamic liver magnetic resonance imaging. Invest Radiol.

[19] Rosenkrantz AB, Geppert C, Grimm R (2015). Dynamic contrast-enhanced MRI of the prostate with high spatiotemporal resolution using compressed sensing, parallel imaging, and continuous golden-angle radial sampling: preliminary experience. J Magn Reson Imaging.

[20] Chen L, Zeng X, Wu Y (2019). A study of the correlation of perfusion parameters in high-resolution GRASP MRI with microvascular density in lung cancer. J Magn Reson Imaging.

[21] Attenberger UI, Liu J, Riffel P (2017). Quantitative perfusion analysis of the rectum using golden-angle radial sparse parallel magnetic resonance imaging: initial experience and comparison to time-resolved angiography with interleaved stochastic trajectories. Invest Radiol.

[22] Riffel P, Zoellner FG, Budjan J (2016). “One-stop-shop”: free-breathing dynamic contrast-enhanced magnetic resonance imaging of the kidney using iterative reconstruction and continuous golden-angle radial sampling. Invest Radiol.

[23] Lim RP, Shapiro M, Wang EY (2008). 3D time-resolved MR angiography (MRA) of the carotid arteries with time-resolved imaging with stochastic trajectories: comparison with 3D contrast-enhanced bolus-chase MRA and 3D time-of-flight MRA. AJNR Am J Neuroradiol.

